# Impact of national policy on hand hygiene promotion activities in hospitals in Korea

**DOI:** 10.1186/s13756-020-00817-3

**Published:** 2020-09-23

**Authors:** Pyoeng Gyun Choe, Jihee Lim, Eun Jin Kim, Jeong Hee Kim, Myoung Jin Shin, Sung Ran Kim, Jun Yong Choi, Young Hwa Choi, Kyung Won Lee, Hyunsook Koo, Hyungmin Lee, Kyoung-Ho Song, Eu Suk Kim, Nam Joong Kim, Myoung-don Oh, Hong Bin Kim

**Affiliations:** 1 Department of Internal Medicine, Seoul National University College of Medicine, 103 Daehak-ro, Jongro-gu, Seoul,, 03080 Republic of Korea; 2 Center for Infection Control and Prevention, Seoul National University Hospital, Seoul, Republic of Korea; 3 Infection Control Office, Seoul National University Bundang Hospital, Seongnam, Republic of Korea; 4 Infection Control Office, Korea University Guro Hospital, Seoul, Republic of Korea; 5 Depart of Internal Medicine, Yonsei University College of Medicine, Seoul, Republic of Korea; 6 Department of Infectious Diseases, Ajou University School of Medicine, Suwon, Republic of Korea; 7 Department of Laboratory Medicine, Yonsei University College of Medicine, Seoul, Republic of Korea; 8 Division of Healthcare Associated Infection Control, Korea Centers for Disease Control and Prevention, Cheongju, Republic of Korea

**Keywords:** Hand hygiene, Policy, Reimbursement, WHO, Infection control practitioners

## Abstract

**Background:**

After the Middle East respiratory syndrome coronavirus outbreak in Korea in 2015, the Government established a strategy for infection prevention to encourage infection control activities in hospitals. The new policy was announced in December 2015 and implemented in September 2016. The aim of this study is to evaluate how infection control activities improved within Korean hospitals after the change in government policy.

**Methods:**

Three cross-sectional surveys using the WHO Hand Hygiene Self-Assessment Framework (HHSAF) were conducted in 2013, 2015, and 2017. Using a multivariable linear regression model, we analyzed the change in total HHSAF score according to survey year.

**Results:**

A total of 32 hospitals participated in the survey in 2013, 52 in 2015, and 101 in 2017. The number of inpatient beds per infection control professionals decreased from 324 in 2013 to 303 in 2015 and 179 in 2017. Most hospitals were at intermediate or advanced levels of progress (90.6% in 2013, 86.6% in 2015, and 94.1% in 2017). In the multivariable linear regression model, total HHSAF score was significantly associated with hospital teaching status (β coefficient of major teaching hospital, 52.6; 95% confidence interval [CI], 8.9 to 96.4; *P* = 0.018), beds size (β coefficient of 100 beds increase, 5.1; 95% CI, 0.3 to 9.8; *P* = 0.038), and survey time (β coefficient of 2017 survey, 45.1; 95% CI, 19.3 to 70.9; *P* = 0.001).

**Conclusions:**

After the new national policy was implemented, the number of infection control professionals increased, and hand hygiene promotion activities were strengthened across Korean hospitals.

## Background

Hand hygiene is critical to the prevention of healthcare-associated infection [1, 2]. In 2013, the National Hand Hygiene Promotion Campaign was launched in Korea with the support of the Korean Centers for Disease Control and Prevention (KCDC) and the Korean Society for Healthcare-associated Infection Control and Prevention. The campaign aims to implement a standardized program for changing hand hygiene based upon the World Health organization (WHO) multimodal hand hygiene improvement strategy. The WHO multimodal strategy is accompanied by an Implementation Toolkit to help the translation to the practice of guideline recommendations [3]. The Implementation Toolkit includes a Hand Hygiene Self-Assessment Framework (HHSAF): a validated tool for evaluating the implementation level of the WHO multimodal hand hygiene improvement strategy [4, 5]. During the Korean campaign, a nationwide survey assessing the use of the Implementation Toolkit was conducted every 2 years.

In 2015, a massive outbreak of Middle East respiratory syndrome coronavirus occurred in Korea; 97% of all cases were healthcare-associated infections [6]. After the outbreak, the Korean Government introduced a policy to enforce the employment of infection control professionals in hospitals. The new Korean Government policy for infection control is developing a novel reimbursement system regarding infection control activities. In order to charge the “infection control fee”, the hospital must fulfill the following criteria: having at least one infection control nurse per 150 inpatient beds; having at least one infection control physician per 300 inpatient beds; participating in Korean Nationwide Healthcare-associated infection surveillance system (KONIS); and providing appropriate education for infection control professionals (> 18 h/year) [7]. The new policy was announced in December 2015 and implemented in September 2016.

This study aims to evaluate how infection control measures improved in hospitals using three cross-sectional surveys conducted during the National Hand Hygiene Promotion Campaign following the change in government policy in Korea.

## Methods

### Participating hospitals

Three cross-sectional surveys were conducted in 2013, 2015, and 2017. All general hospitals and hospitals with more than 150 beds operating infection control offices were eligible for National Hand Hygiene Promotion Campaign in Korea. In the 2013 and 2015 survey, all hospitals participated in the National Hand Hygiene Promotion Campaign were included. In the 2017 survey, all hospitals registered for the KONIS were invited via e-mail to voluntarily participate in the survey.

### Survey

The HHSAF is a questionnaire comprising 27 items and is grouped into five sections that reflect the WHO Multimodal Hand Hygiene Improvement Strategy (namely, system change, training and education, evaluation and performance feedback, reminders in the workplace, and institutional safety climate) [5]. Each component is scored out of 100 points (total maximum score: 500). According to their overall score, health care facilities are assigned to 1 of 4 levels for the hand hygiene implementation progress: inadequate (score of 0–125), basic (score of 126–250), intermediate (score of 251–375), or advanced (score of 376–500) [5]. Participants were also asked to provide information regarding the characteristics of their hospital including; facility type (i.e., public or private sector, general or teaching status); the number of inpatient beds; the number of full-time equivalent infection control professionals and physician epidemiologists.

### Statistical analysis

Descriptive results of continuous variables were expressed as median values and interquartile range (IQR). A multivariable linear regression model was used to analyze the change in the number of inpatient beds per infection control professionals and total HHSAF score after adjusting for hospital characteristics. The model included hospital type (public or private sector), hospital teaching status, number of hospital beds, and survey year. We calibrated the correlations for hospitals that are repeatedly measured by multiple observations using a generalized estimated equation. All significance tests were 2-sided, and data analysis were performed using Stata software (version 15.1; StataCrop LLC., College Station, TX).

## Results

### Hospital characteristics

A total of 286 hospitals were eligible for the National Hand Hygiene Promotion Campaign and candidates for the survey in 2013, 319 in 2015, and 388 in 2017. Of these eligible hospitals, 32 (11.2%) hospitals participated in the survey in 2013, 52 (16.3%) in 2015, and 101 (26.0%) in 2017. Characteristics of the participating hospitals are shown in Table 1. Most hospitals were private sector hospitals (71.9% in 2013, 71.1% in 2015, and 74.3% in 2017). Small size hospitals and non-teaching hospitals participated more in 2017; the proportion of hospitals which were major teaching hospitals were: 84.4% in 2013, 67.3% in 2015, and 39.6% in 2017. The median number of beds per hospital was 820 (IQR, 641–953) in 2013, 698 (IQR, 377–864) in 2015, and 545 (IQR, 287–767) in 2017.

**Table 1:** The characteristics of participating hospitals according to the survey year

	2013	2015	2017
Total n. of eligible hospitals	286	319	388
Total n. of participating hospitals (n, %)	32 (11.2)	52 (16.3)	101 (26.0)
Hospital type (n, %)			
Public	9 (28.1)	15 (28.9)	26 (25.7)
Private	23 (71.9)	37 (71.1)	75 (74.3)
Teaching status (n, %)			
Major teaching hospitals	27 (84.4)	35 (67.3)	40 (39.6)
Minor teaching hospitals	5 (15.6)	11 (21.2)	27 (26.7)
Nonteaching hospitals		6 (11.5)	34 (34.7)
Beds per hospitals (median)	820	698	545
Infection control professional per hospital (median)	2.5	2.0	3
Beds per infection control professional (median)	309	281	178

### The number of inpatient beds per infection control professionals

The median number of inpatient beds per infection control professionals decreased from 324 in 2013 to 303 in 2015 and 179 in 2017 (Table 1). In multivariate linear regression analysis, the number of inpatient beds per infection control professionals was not associated with hospital types and teaching status (Table 2). In the model, the number of inpatient beds per infection control professionals decreased by 19.6 in 2015 (*P* = 0.148) and 141.9 in 2017 (*P* <  0.001).

**Table 2:** Multivariable linear regression analysis for the inpatient beds per infection control professionals

	β coefficient	95% Confidence interval	*P*-value
Hospital type			
Public (ref)			
Private	12.9	−15.7 to 41.5	0.378
Teaching status			
Nonteaching hospital (ref)			
Minor teaching hospital	8.3	−31.3 to 41.9	0.681
Major teaching hospital	10.5	−21.6 to 42.5	0.523
Survey year			
2013 (ref)			
2015	−19.6	−46.2 to 7.0	0.148
2017	−141.9	− 181.7 to − 102.2	< 0.001

### HHSAF results

**Fig. 1:**
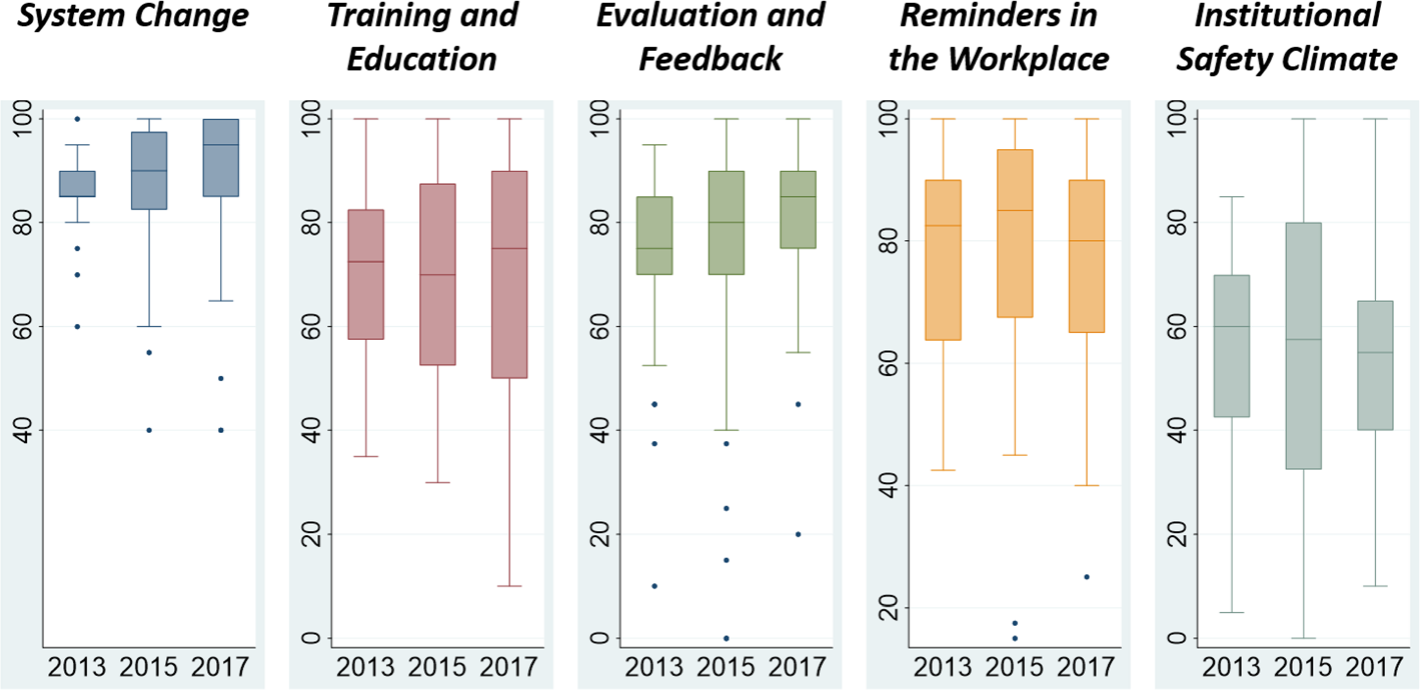
WHO Hand Hygiene Self-Assessment Framework scores by category

According to the HHSAF score, most hospitals were at intermediate or advanced levels of progress in implementing hand hygiene protocols (90.6% in 2013, 86.6% in 2015, and 94.1% in 2017). The median total HHSAF scores were 375 (range, 162–450) in 2013, 397.5 (range 135–475) in 2015, and 375 (range 145–480) in 2017, which reflects an advanced level of progress across all surveys (Table 3). Among HHSAF sections, the highest score was for the system change, and the lowest scores were for institutional safety climate (Fig. 1.).

**Table 3:** Overall WHO Hand Hygiene Self-Assessment Framework score and level in participating hospitals according to the survey year

	2013	2015	2017
Total n. of hospitals (n, %)	32	52	101
Overall score, median (range)	375 (162–450)	397.5 (135–475)	375 (145–480)
Hand hygiene level, n (%)			
Inadequate	0	0	0
Basic	3 (9.4)	7 (13.4)	6 (5.9)
Intermediate	8 (25.0)	13 (25.0)	34 (33.7)
Advanced	21 (65.6)	32 (61.5)	61 (60.4)

In multivariable linear regression model, total HHSAF score was significantly associated with hospital teaching status (β coefficient of major teaching hospital, 52.6, 95% confidence intervals, 8.9 to 96.4, *P* = 0.018), and beds size (β coefficient of 100 beds increase, 5.1, 95% confidence intervals, 0.3 to 9.8, *P* = 0.038). In the model, the estimated total HHSAF score increased by 20.2 in 2015 (*P* = 0.065) and 45.1 in 2017 (*P* = 0.001) (Table 4).

**Table 4:** Multivariable linear regression analysis for the WHO Hand Hygiene Self-Assessment Framework total score

	β coefficient	95% Confidence interval	*P*-value
Hospital type			
Public (ref)			
Private	7.1	−16.3 to 40.5	0.552
Teaching status			
Nonteaching hospital (ref)			
Minor teaching hospital	19.9	−17.1 to 56.8	0.292
Major teaching hospital	52.6	8.8 to 96.4	0.018
Beds size (per 100 increase)	5.1	0.3 to 9.8	0.038
Survey year			
2013 (ref)			
2015	20.2	−1.0 to 41.4	0.062
2017	45.1	19.3 to 70.9	0.001

## Discussion

Having an advanced level in terms of implementing the hand hygiene program is associated with the level of infection control staffing, in particular, to achieve reasonable progress in education and for creating an institutional safety climate. The SENIC Project (Study on the Efficacy of Nosocomial Infection Control) calculated the adequate ratio of one infection control professional per 250 hospital beds more than 30 years ago [8]; this ratio was used as a reference for a long period of time. The Delphi project conducted in 2001, suggested that a ratio of 0.8–1 infection control professionals per 100 hospital beds may be needed to effectively drive improvement [9]. This is also supported by recent studies [10, 11].

In Korea, the median number of inpatient beds per infection control professionals among the hospitals which participated in KONIS in 2006 was 580; this was maintained above 300 until 2015 [12]. This was twice as high as the number in US hospitals [13]. The ratio remarkably changed after the infection control policy was introduced by the Korean Government in 2016. The median number of inpatient beds per infection control professional was 141 among hospitals which participated in KONIS in 2018. In this survey, the median number of inpatient beds per infection control professional was 309 in 2013 and 289 in 2015 and markedly decreased to 178 in 2017; this was significant in multivariable linear regression analysis. As a result, the degree of infection control activity measured by the WHO HHASF significantly improved in Korean hospitals.

The compliance of hand hygiene also improved across Korean hospitals. Since 2013 the National Hand Hygiene Promotion Campaign instigated by KCDC conducted several pilot surveillances for hand hygiene compliance in hospitals [14]. Although a simple comparison is difficult as the participating hospitals are different during each surveillance period, hand hygiene compliance was 67.2% in 35 hospitals between November 2013 and February 2014, which increased to 83% in 23 hospitals between September 2016 and January 2017, and 85.2% in 61 hospitals between February 2018 and June 2018.

Our surveys, especially the 2017 survey, presents a snapshot of the current level of implementation of hand hygiene improvement programs in Korean healthcare facilities. In the 2017 survey, most hospitals (94.1%) were at intermediate and advanced levels of progression; there were no hospitals with inadequate levels. Based on the HHSAF, the median score in the 2017 survey indicated that the level of progress was at an “advanced” level. These results mean that an appropriate hand hygiene promotion strategy is in place and the practices have improved in Korea, which was similar to the US survey for 168 facilities conducted in 2011 and the global survey for 86 facilities conducted in 2015 [11, 15].

In the 2017 survey, specific component scores were higher for system change (median 95, IQR 85–100) and evaluation and feedback (median 85, IQR 75–90). These scores had significantly improved compared to previous surveys. This result suggested that the facilities for hand hygiene, such as easy access to alcohol-based hand sanitizer, have been sufficiently improving and the activities for hand hygiene monitoring and feedback have been well established in Korean hospitals. The institutional safety climate around hand hygiene was the element of the strategy that scored the lowest in all surveys (median scores, 55–60), which did not significantly change over time. In both US and global surveys, the score for institutional safety climate was also the lowest [11, 15]. This likely reflects the challenges to convey the concept that infection control and preventive interventions can be significantly enhanced when understood in the context of a positive safety culture.

In this study, total HHSAF score was significantly associated with hospital size and teaching status. In contrast to our results, there was no association between total HHSAF score and facility size and teaching status in the survey conducted in the US [11]. This suggested that the infection control activities in Korea have been conducted mainly at large teaching hospitals and have not sufficiently permeated through to smaller sized hospitals.

Our study has several limitations. First, although the usability and reliability of the WHO HHSAF was well established [4], reporting bias may be present as the results rely on self-assessment. Second, this study results might exaggerate the actual status of hand hygiene activities in Korea because hospitals voluntarily participated in the surveys and hospitals with more hand hygiene promotion activities may have participated in the survey.

## Conclusion

In conclusion, after the national policy implementation, the number of infection control professionals increased, and promotion of hand hygiene activities were strengthened in Korean hospitals.

## Data Availability

The datasets used and analyzed in the context of this survey are available from corresponding author upon reasonable request.

## Abbreviations

HHSAF: Hand Hygiene Self-Assessment Framework
KCDC: Korea Centers for Disease Control and Prevention
KONIS: Korean Nationwide Healthcare-associated infection surveillance system
IQR: Interquartile range
WHO: World Health organization
